# Acknowledgment

**Published:** 1842-03

**Authors:** C. A. Harris

**Affiliations:** Cor. Sec. A. S. D. S.


*Acknowledgment.*—Received from the author for the American Society of Dental Surgeons, *Three Memoirs on the Development and Structure of Teeth and Epithelium,* read at the ninth annual meeting of the British Association for the encouragement of science, held at Birmingham, in August, 1839, with diagrams exhibited in illustration of them. By Alexander Nasmyth, F. L. S., F. G. S. member of the Royal College of Surgeons, &c. London, 1841. C. A. Harris, *Cor. Sec. A. S. D. S.*

